# Strengthening the WHO in the pandemic era by removing a persistent structural defect in financing

**DOI:** 10.1186/s12992-021-00780-7

**Published:** 2021-12-15

**Authors:** Nkuchia M. M’ikanatha, David P. Welliver

**Affiliations:** 1 Center for Clinical Epidemiology and Biostatistics, Perelman School of Medicine, University of Pennsylvania, Philadelphia, PA 19104 USA; 2 Clarific Services, Columbus, OH 43220 USA

**Keywords:** World Health Organization, Global health, Financing, Pandemics, International health regulations, Communicable diseases, Healthcare reform

## Abstract

**Background:**

The WHO’s success in its vital role is constrained by inadequate financial support from member states and overreliance on earmarked voluntary contributions, which erodes autonomy. The agency’s broad functions, including coordination among 194 members, cannot be performed by any other entity. However, despite experts’ well-articulated concerns that the agency’s legitimacy and authority in global health matters have been undermined, a decades-long freeze on member assessments means that WHO priorities are disproportionately influenced by a few powerful donors.

**A structural defect:**

To overcome inertia in addressing well-known limitations, it may be helpful to consider the weaknesses in WHO’s financing mechanism as a persistent *structural defect*. This perspective strengthens the focus on corrections needed to remove the defect. In our view, the main features of the structural defect are the self-imposed constraints that foster the perception—if not the reality—that the agency’s legitimacy is compromised. These constraints include WHO’s inadequate level of financing; lack of direct control over 80% of its funds; and unbalanced participation, such that over 60% of financing originates from only 9 donors. With renewed commitment, however, member countries can remove these constraints.

**Removing the structural defect:**

To meaningfully strengthen structural integrity of the financing mechanism, restore WHO’s autonomy, and minimize concerns about wealthy-donor supremacy, it will be necessary to define specific requirements and implement restrictions on financial contributions. We make five recommendations, including tripling total financing; ensuring that 70% or more of financial support derives from member assessments; limiting contributions from individual members to a maximum of 4% of total WHO financing; and limiting donations from individual partners to a maximum of 3% of total WHO financing (1% for earmarked donations). Although some might consider these measures impractical, they are justified by the magnitude of the crises the world faces, by member states’ increased economic strength in recent decades, and by the importance of shielding the WHO’s financing structure from perceived neocolonialism. This necessary step calls for an adjustment of priorities: the higher level of assessed contribution—from nearly all members regardless of wealth—required to reach the proposed targets would still represent only a small fraction of most members’ annual military expenditures.

**Conclusion:**

The COVID-19 pandemic, with its devastating toll on human life and global economic stability, presents an opportunity for reflection and refocusing. Realigning WHO’s financial structure to its founders’ vision, as proposed here, would likely safeguard both the agency’s autonomy and member states’ trust, while alleviating concerns about undue influence from powerful donors. Removing the persistent structural defect in financing would empower WHO to lead and coordinate global response to meet the inevitable challenges of the coming decades.

**Supplementary Information:**

The online version contains supplementary material available at 10.1186/s12992-021-00780-7.

## Background

The coronavirus disease 2019 (COVID-19) pandemic brings focus to global public health and the institutions designed to safeguard it. As of 24 September 2021, approximately 230.4 million confirmed cases of COVID-19 and 4.7 million deaths had been reported [1]. The World Health Organization (WHO) plays a unique role in declaring public health emergencies of international concern (PHEICs) and coordinating timely international response. The agency’s scope of responsibility has increased considerably in recent years, not only because of infectious disease threats such as severe acute respiratory syndrome (SARS), Ebola, and COVID-19, but also in response to the expanding global impact of chronic diseases, substance abuse, mental disorders, obesity, malnutrition, and many other public health concerns. In fact, to meaningfully address the threat of antimicrobial resistance alone may require as much of the WHO’s attention and coordination as does the current pandemic. These public health challenges have been exacerbated by the interplay of factors including climate change, population movement, economic disparities, and changes in behavioral patterns. Concerning climate change, widespread alarm about the increasing intensity of its effects—including severe public health consequences—is evidenced by the recent appearance in leading health journals across the world of an editorial calling for urgent changes and more attention to global coordination [2].

However, at this time when a strong WHO is so vitally needed, controversy has arisen regarding the effectiveness of the agency, and many have proposed reforms. In large part, concerns are rooted in a perception that the WHO lacks autonomy and thus cannot act decisively in a crisis such as the current pandemic. Calls to restore the agency’s legitimacy and full authority in global health matters underscore the severity of the problem [3].

While elected officials and policy makers in some countries have criticized the agency and argued for withdrawing financial support, many public health specialists have viewed the problem differently, pointing to an insufficient level of financing. Specifically, they caution that the WHO is susceptible to undue influence from powerful members and external donors because of its financial vulnerability. Here, we offer a perspective on recent discussions about the agency’s financial sustainability, identify a persistent structural defect in the WHO’s financing mechanism, and propose steps to remedy the defect as a prerequisite to addressing any other called-for reforms.

Severe acute respiratory syndrome coronavirus 2 (SARS-CoV-2) emerged at the end of a decade in which alarms had already been raised about financial constraints that hamper the WHO’s ability to meet its priorities, and this situation has generated intense discussion. For example, in a 2018 literature review on the financial sustainability of the WHO, Reddy et al. distilled previous recommendations from 56 peer-reviewed articles to help clarify the role and future of the agency. The authors identified areas where there is consensus for reform, such as the need for a contingency fund for emergencies, as well as areas where disagreements remain, in particular with respect to changes in the assessed member contributions (dues) to fund the agency’s mandated responsibilities [4]. The review highlights the fact that in the early 1980s the World Health Assembly (WHA) voted to freeze assessed member contributions; then in 1993 the WHA took the further step of eliminating adjustments for inflation and currency fluctuation. Consequently, in the ensuing years, even while the agency’s responsibilities have evolved in scope and complexity in parallel to rising global challenges, the WHO has become dependent on voluntary contributions. The WHO leadership has increasingly emphasized the dangers inherent in such dependency, for example at the most recent WHA session (May 2021), when—against the backdrop of a relentless pandemic—the Director-General called for “a paradigm shift in the quantity and quality of funding” and warned that “WHO cannot grow stronger without sustainable financing” [5]. Although the WHO’s budget is meant to reflect priorities set by the WHA, this structure has been distorted by overreliance on earmarked contributions: some of the agency’s priorities have attracted such contributions, but it should be acknowledged that many of the WHO’s core activities have elicited little interest from donors and, as we point out later, some are even actively opposed by voluntary contributors.

By 1994 it was clear that the preferred method for countries and other donors making voluntary contributions was to earmark the funds for freestanding programs within the WHO, programs with dedicated management committees that included donor representation. This practice, while appealing to some member states, rendered the WHO “increasingly dispersed and uncoordinated” and brought the criticism that “WHO’s priorities now reflect donors’ preferences rather than rational allocation of resources” [6]. Today, a quarter of a century later, this undesirable situation has not changed, except that external donors now play a much larger role. With the longstanding ceiling on member assessments, and no corresponding limit to voluntary earmarked contributions from either member states or external donors, the proportion of WHO financing outside the agency’s direct control has now reached 80%, with 53% of total financing coming from non-member sources [7]. Continuing dependence on earmarked contributions, as global health experts have pointed out, “creates a situation where external donors dictate the organization’s priorities and action agenda” [4, 8]. A shift in priorities can also occur when member states making large contributions exert their influence by actively opposing the development of evidence-based policies for political expediency, as has been observed in the past with guidelines on sugar content in food, tobacco control, breast-milk substitutes, and ethical marketing of medical products. Furthermore, as the world has witnessed during the last decade, derailing the agency in this way means that all countries are now more vulnerable to PHEICs and their inevitable toll in human life and economic loss.

## Financing mechanism must support WHO’s unique role

Inclusivity and uninhibited participation are central to the WHO’s mission. In recent years, and especially during the COVID-19 pandemic, leaders in global public health and in international affairs have persuasively called upon all countries to renew their commitment to the WHO, to its principles, its technical leadership, and its coordinating role. One such plea, for example, while acknowledging the WHO’s imperfections, emphasizes the need for global solidarity in strengthening the agency and urges member states to be fully engaged in the implementation of recommended measures, including timely reporting of epidemiological data [3].

The broad functions of the WHO cannot realistically be performed by any other entity. They require centralized coordination by an agency whose authority to act is voluntarily bestowed by sovereign nations. These functions are premised on a collective understanding that all member countries will implement certain programs and activities, as well as participate in standardized assessment of global status by the centralized agency [9].

By creating a financial structure in which responsibility would be equitably distributed among all member states, taking into account each member’s relative wealth and population, the founding WHA was intentional in safeguarding both the agency’s autonomy and member states’ trust. The unique role of the WHO as a leading and coordinating body acting on behalf of sovereign states demanded that its financial resources be predictable and within its own control. However, as others have observed, the original safeguards are no longer in place and, as a consequence, the WHO’s financial resources are unpredictable, the agency is overly reliant on voluntary contributions, its agenda is largely externally dictated, and its legitimacy as leader in global health matters is undermined [10, 11].

Like its parent organization, the United Nations, the WHO can be successful in its mission only when its members accept their interdependence and prioritize collective well-being over narrow self-interest. In our view, perilous global conditions make it imperative to foster genuine collaboration and unity among member states, and a shared vision of transformative collaboration can only be achieved if courageous steps are taken to remove the power imbalance that inevitably accompanies an extreme imbalance in financing participation. Addressing the paramount necessity for unity, inclusivity and collaboration at the level of the United Nations, an insightful recent commentary makes the point in more emphatic terms: “Objectives incompatible with the pursuit of the common good will need to be set aside” and “… failure to reach an arrangement supporting effective global coordination risks consequences far more severe—potentially catastrophic—than those arising from recent disruptions. The task before the community of nations, then, is to ensure that the machinery of international politics and power is increasingly directed toward cooperation and unity” [12].

## A structural defect

The weaknesses in the WHO’s financing mechanism that constrain the agency’s effectiveness are well-known and largely self-imposed, yet the will to remove these constraints has been lacking. To overcome inertia, it may be helpful to consider these weaknesses as a persistent *structural defect*. This perspective permits a strengthened focus on the corrections needed to remove the defect. In our view, the main features of the structural defect are the constraints that foster the perception—if not the reality—that the agency’s legitimacy is compromised. These constraints include the WHO’s inadequate level of financing; lack of direct control over 80% of its funds; and unbalanced participation, such that over 60% of financing originates from only 9 donors. The fact that the largest contributions to the WHO are voluntary and therefore inherently unpredictable amplifies the defect.

**Fig. 1:**
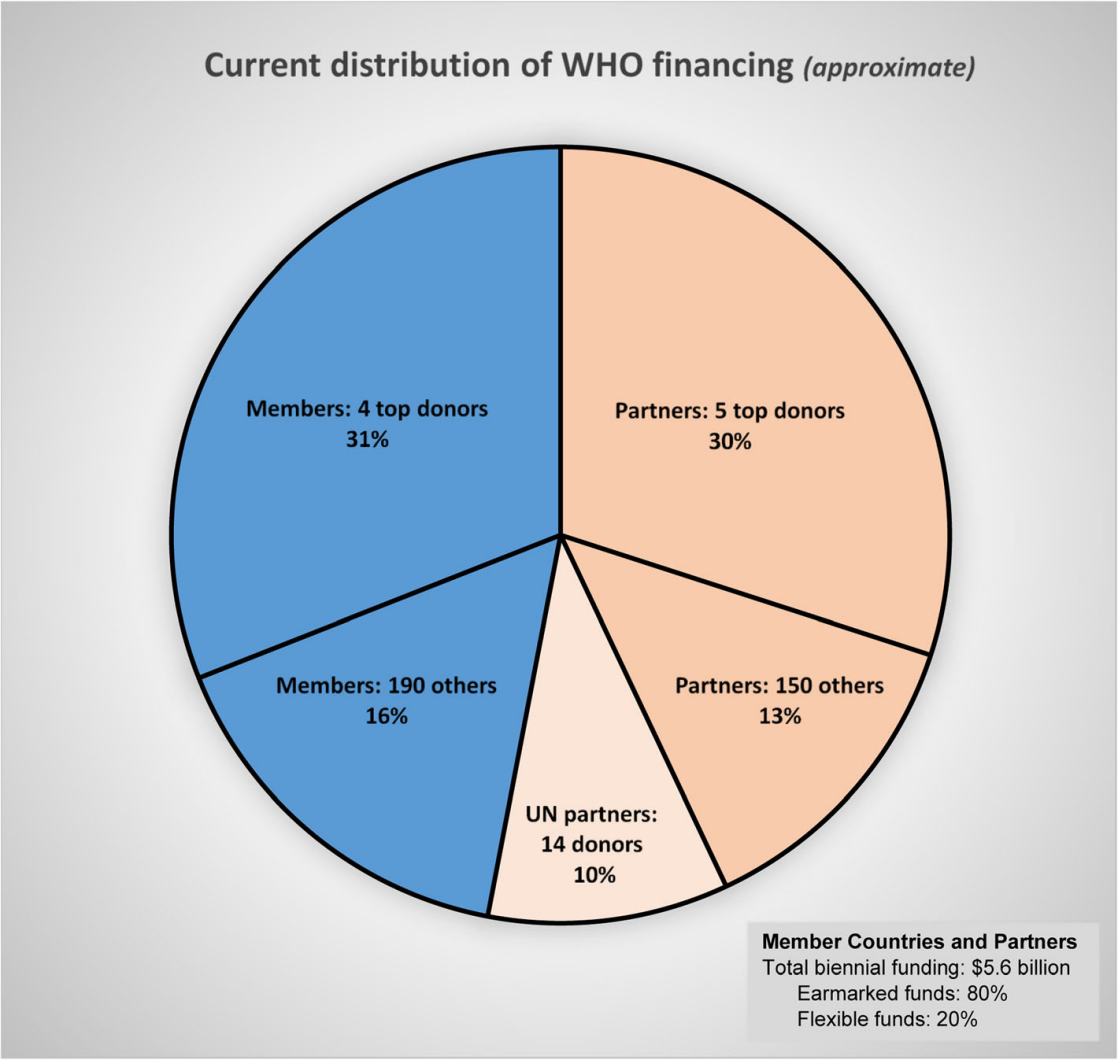
Current WHO financing (approximate) as distributed among member countries and external partners

In the 2018–2019 biennium, only 20% of the WHO’s $5.6 billion funding came from “flexible” contributions, consisting of member assessments and other contributions having no earmarks to constrain their use. The remaining 80% is under the category of “specified” contributions, and the donor directs how the money will be used [7]. In terms of breadth of support, approximately 31% of the WHO’s financing is shouldered by only four member countries, with 16% being given by the other 190 member countries; the remaining 53% is contributed by external partners, with just 5 of these partners accounting for 30% of the WHO’s total financing. (See Fig. 1.)

This financing structure significantly departs from what was originally envisioned and outlined in the WHO’s constitution. As explained above, the current structure developed as voluntary contributions began to take a dominant role. It is hard to imagine that the present situation is what the founders of the WHO intended.

## Removing the structural defect

Scholars have previously suggested various ways to address one or more of the shortcomings in the current financing structure. For example, in 2015 one expert proposed doubling the agency’s financing and argued that 50% of WHO financing should come from member assessments [11]. No action on such proposals has so far been taken. Considering that the world has experienced a catastrophe with COVID-19 that is still unfolding, and that preparation for future crises will be desirable, it is an opportune moment for the WHA to remove the persistent structural defect in financing.

To empower the WHO to fulfill its mission, it will be essential to address not only the overall level of financing but all of the component parts that make up the defect. Specifically, removal of the structural defect in financing will involve changes in the level, predictability and sources of WHO financing, and must ensure equitable distribution of the financing responsibility across all member states. To ensure that these changes will be implemented in a way that best strengthens structural integrity of the financing mechanism, we are convinced that it will be necessary to define specific requirements and implement restrictions on financial contributions. While there may be other ways to rectify the problem, in consideration of the insightful provisions of the WHO constitution the following are perhaps the most direct and appropriate means through which the WHA can restore structural integrity to the agency’s financing.

**Table:** 

**How to remove the structural defect in WHO financing**	
1. Increase biennial financing to $US 16.8 billion—three times the current level—a level commensurate with the WHO’s responsibilities.	
2. Ensure that the bulk of financial support (ideally 70% or more) derives from assessments to member countries, as was originally envisioned in the WHO’s constitution.	
3. Preserve WHO autonomy by transitioning nearly all voluntary contributions from the *earmarked* to the *flexible* category.	
4. Ensure that donations from member states are equitably distributed so as to avoid undue influence from any one country: Limit contributions from individual members to a maximum of 4% of total WHO financing.	
5. Realign WHO’s financing with its constitutional mandate by limiting external donations: Limit donations from individual partners to a maximum of 3% of total WHO financing (1% for earmarked donations).	

### 1. Increase total financing to a level commensurate with WHO’s responsibilities

As noted above, it has been acknowledged by members, and pointed out by scholars, that the current level of WHO financing is insufficient for the agency to fulfill its responsibilities and that something must be done to address the mismatch between financing and the scope of work. Considering infectious disease surveillance alone, though only one of the WHO’s many areas of concern, it is clear that this function will continue to evolve in complexity. Challenges to surveillance including—but not limited to—new pathogens, climate change, natural disasters, travel, population displacement, political instability, poverty, and globalization of food production will bring new crises, necessitating stronger collaboration and information sharing among local, regional, national and international participants [13]. Only the WHO is positioned to coordinate and foster alignment across all these levels.

With 7.9 billion people on the planet, the WHO’s biennial funding of $5.6 billion constitutes an expenditure of only $0.71 per person. We suggest that an increase from the current level to the modest amount of $2.13 per person would go far toward strengthening the agency and improving global health. Such an increase, resulting in approximately $16.8 billion in biennial financing—three times the current level—would demonstrate commitment from the member states and would most likely lead to more productive engagement of members with the WHO.

This level of investment is reasonable also from a purely economic perspective. Considering the amount that must be spent in containing a PHEIC, for example, even a modest increase in investment in the WHO will likely result in significant cost avoidance for member countries while yielding a high return in global public health. During the present pandemic, trillions of dollars have already been spent by member countries to avert human suffering and economic devastation that would almost certainly have been reduced if the WHO had been less constrained in fulfilling its responsibilities. Furthermore, the threat of pandemics is not the only challenge requiring strong WHO leadership and commensurate financing.

Most importantly from the perspective of systemic integrity, an increase in WHO financing is necessary to fully remove the structural defect because unmet financial needs inevitably make the agency vulnerable to dependence on earmarked donations, thereby weakening its autonomy and effectiveness.

### 2. Ensure that financial support derives primarily from assessments to member countries, as was originally envisioned in the WHO constitution

WHO member states naturally have a strong interest in ensuring a predictable, uninterrupted flow of funds as well as minimizing any disproportionate influence from powerful non-member donors. In our view, these important objectives can best be achieved if member states provide through assessed contributions at least 70% of total WHO financing. This correction will only be possible if the WHA closely monitors all contributions so that they stay within specified proportions, as is further elaborated in recommendations 4 and 5 below. This approach would be consistent with the way in which the WHO was financed in its early years, and would minimize concerns about negative influence.

### 3. Preserve WHO autonomy by transitioning nearly all voluntary contributions from the earmarked to the flexible category

To further strengthen the WHO’s control over its own funds, voluntary (non-assessed) contributions should ideally be designated as *flexible* (“unspecified”) donations rather than *earmarked* (“specified”) donations. Additionally, to be consistent with recommendation 2 above, the voluntary donations from all members combined should not exceed 30% of total WHO financing.

Earmarked contributions have elicited scrutiny and debate among member states because of the potential for real or perceived influence from donors. This influence could occur for example through privileged access to technical staff or to priority-setting mechanisms, providing a donor the ability to shift the WHO’s priorities or alter its implementation processes. The correction in specification of donor funds proposed here addresses concerns expressed by member states regarding preservation of member supremacy and limiting the WHO’s dependence on external entities [4].

### 4. Ensure that donations from member states are equitably distributed so as to avoid undue influence from any one country, and to promote broad support

This correction is needed because at present, as noted above, the greater part of WHO financing is shouldered by only a handful of donors. Allowing a member country to contribute a disproportionately high amount in comparison to others introduces the reality or perception of undue influence.

Equally important is the need for all members to actively collaborate with the WHO; we underscore, as previously mentioned, that the commitment of all members is vital, and a structure where financial responsibility is equitably distributed most clearly demonstrates and preserves stakeholder commitment.

To implement this change, we recommend limiting the total contribution (assessed plus voluntary) from any individual member state to 4% of total WHO financing. Breaking this down further, we suggest setting per-donor limits of 2% for the assessed category, 2% for the voluntary flexible category, and 1% for the voluntary specified (earmarked) category. Such limits take into consideration the need to balance broad distribution of financial responsibility with a fair apportioning of assessments according to each country’s population and financial capacity. This correction should not be viewed as a drastic measure: If we assume 70% of total WHO financing were to come from member assessments (in accordance with recommendation number 2 above), then an equal—though not equitable—percentage contribution from each of the 194 members would come to 0.36% of total WHO financing being assessed to each member. By distributing actual assessments between 0.0 and 2.0% of total WHO financing (with a mean of 0.36%), taking into consideration each member’s population and financial capacity, the objective would be achieved. In addition, opportunities for voluntary contribution within the specified limits would still remain.

### 5. Realign WHO’s financing with its constitutional mandate by limiting the proportion of total financing contributed by any one non-member partner

When non-state donors make large contributions to the WHO, however well-intentioned, confidence in the agency’s governance and impartiality is potentially undermined. As pointed out above, the constitution specifies that the agency’s budget is to be funded by assessments to the member states; gifts were intended to play a minor role, and were to be accepted only if “consistent with the objective and policies of the Organization” [14]. When significant support is received through philanthropic sources, it confers prestige and can grant favored access, which results in real or perceived negative influence. Such an ever-present cloud hovers at all levels, ranging from WHA forums where critical decisions about the mission of the agency are made, to the local level where programs are implemented.

To avoid this scenario, we propose that donations from any individual non-member partner should be limited to a maximum of 3% of total WHO financing. This contribution should ideally be *unspecified* (flexible), to conform with recommendation 3 above. However, if an earmark is desired by the donor and is consistent with WHO objectives, we suggest limiting the earmarked amount to a maximum of 1% of total WHO financing.

This correction, in combination with the others discussed above, rectifies the structural defect in the WHO’s financing mechanism by ensuring that the majority of the budget is covered by the member states. The change also reduces vulnerability by minimizing the risk that a change in a partner’s priorities would destabilize the WHO’s predictable sources of funding. Furthermore, it is in line with the expectations of some member countries that have emphasized, as mentioned above, the importance of honoring member states’ supremacy and have called for caution in interactions with non-state actors [4].

**Fig. 2:**
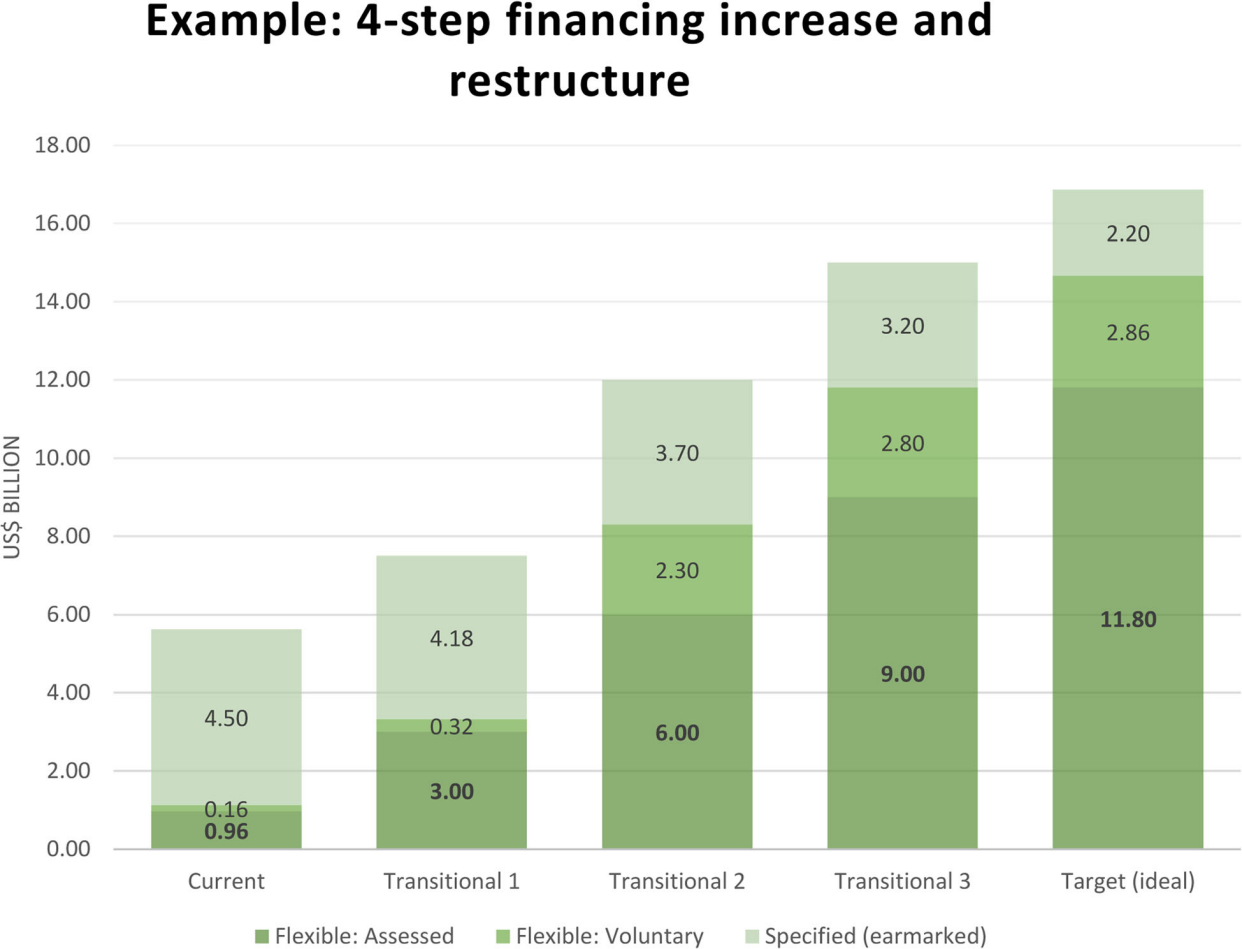
Example of a multi-step model for restructuring and increasing WHO financing. Flexible funds (assessed and voluntary) increase over time, while earmarked funds transition to the flexible voluntary category

It is important to note that the corrections described in the five recommendations above must be considered together as parts of a whole, constituting a complete remedy for removing the structural defect in financing that currently impacts the WHO’s effectiveness. On a practical level, there may be many alternatives for applying these corrections, including various percentage limits and timeframes for implementation. Here, we provide an example of a multi-step financing model whereby the objectives could be met over a period of time (see Fig. 2). Created using a spreadsheet-based tool *(see supplementary material)*, this example illustrates a 4-step approach that gradually increases total member assessments while simultaneously decreasing total earmarked contributions to achieve specified contribution levels while remaining within desirable structural limits.

## Are the proposed corrections unrealistic?

At first glance the proposed financing structure might appear to be idealistic or impractical because it prescribes a significant increase in assessed contributions, even for low- and middle-income member states, while restricting the contribution from any one member to a small fraction of the total. While it seems easier to allow some countries to subsidize others without restrictions, this approach does not strengthen the WHO. The WHO is not an international aid organization; yet its current financing structure resembles that of agencies having economic, political or humanitarian motivations whose mission is to provide assistance to countries deemed “less developed” by wealthy nations using their own measures. By failing to distinguish itself from such agencies in its financing structure, the WHO is vulnerable to growing criticisms that question real or perceived neocolonialist and supremacist patterns and warn against their detrimental effects on global health policy and practice [15]. As underscored above, global public health challenges are expected to increase in intensity in an era of pandemics, climate change and the growing threat of antimicrobial resistance. Meeting these challenges will require an unprecedented level of unity and trust among countries: thus, we need a WHO that is meaningfully supported by all its members without some being under the shadow of others. A broader base of member financial support promotes inclusivity rather than hierarchy.

A significant increase in assessed contributions from nearly all members is not unrealistic. As shown in Table 1, where WHO members are grouped according to the current level of their assessed contributions, economic performance over the past two decades has improved for all assessment-level groups, with per-capita gross domestic product increasing at least 47% (group A) and as much as 91% (group C). During the same period, member assessment-level groups increased their military expenditures by as much as 106% (group C). However, despite growth in economic strength, members’ assessed contributions to the WHO remain as low as 0.01% of military expenditures (group B) and no higher than 0.03% of military expenditures (groups D and E). The COVID-19 pandemic has clearly demonstrated that microbes can cause social and economic devastation in all countries irrespective of their military might. The myriad threats facing humanity demand reprioritization in all countries, and it is worth noting that national security strategists in some nations are beginning to widen the scope of their concerns by adding climate change and public health matters, including pandemic preparedness, to their conventional military considerations.

**Table 1:**
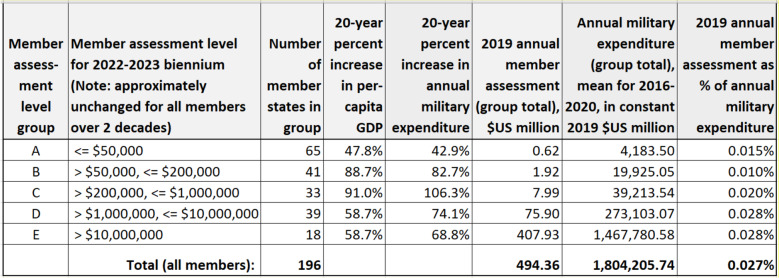
Member states’ financial support to the WHO in relation to their military expenditures: assessments remain low despite two decades of economic growth

**Data sources:** Member assessment data are from World Health Organization ‘Funding by contributor’ datasets [7]. World Bank datasets were used to calculate percentage increases in per-capita GDP (constant $US, 2010 equivalence) [16]. Military expenditure data are from the SIPRI Military Expenditure Database (constant $US, 2019 equivalence) [17]

**Table 1 notes:**

1. Member assessment level groups (A-E) were created by the authors as a convenient way to summarize data. The data summarized here represent 194 WHO members and 2 associate members. *(See supplementary material for a list of countries within each group)*

2. 20-year percentage increases in per-capita GDP are means of member country increases/decreases within each assessment-level group

3. WHO member assessment figures and military expenditure figures are sums of member country amounts within each assessment-level group (in $US millions)

4. Figures are imprecise because we created group-level values by summing individual-country values that were rounded in the source datasets in some cases. In addition, we calculated means over a 4-year period (with per-capita GDP data) and over a 5-year period (with military expenditure data) to account for year-to-year variation and occasional missing values

Table 2 illustrates a scenario in which the recommendations pertaining to assessed contributions have been followed to restore structural integrity to WHO financing. In this scenario total financing is $US 8.4 billion annually, which is triple the 2018–2019 level (recommendation 1); member assessments make up 70% of total financing (recommendation 2); and each member’s assessment is restricted to no more than 2% of total financing (recommendation 4). The illustration balances these requirements with the need to respect differences in financial capacity among the various member groups (A-E). As Table 2 demonstrates, the necessary increase in member assessments for each group, though substantial, nevertheless results in an assessment that equates to only a small fraction (less than 1 %) of annual military expenditures. At the same time, the illustration shows that the proportional share of funding contributed by the handful of wealthiest members can be greatly reduced so long as all other members participate at the suggested assessment levels. The example scenario demonstrates that the structural defect we have described can be removed by giving all members an opportunity to meaningfully strengthen the WHO, but this requires a united resolve on the part of the WHA to finally change course.

**Table 2:**
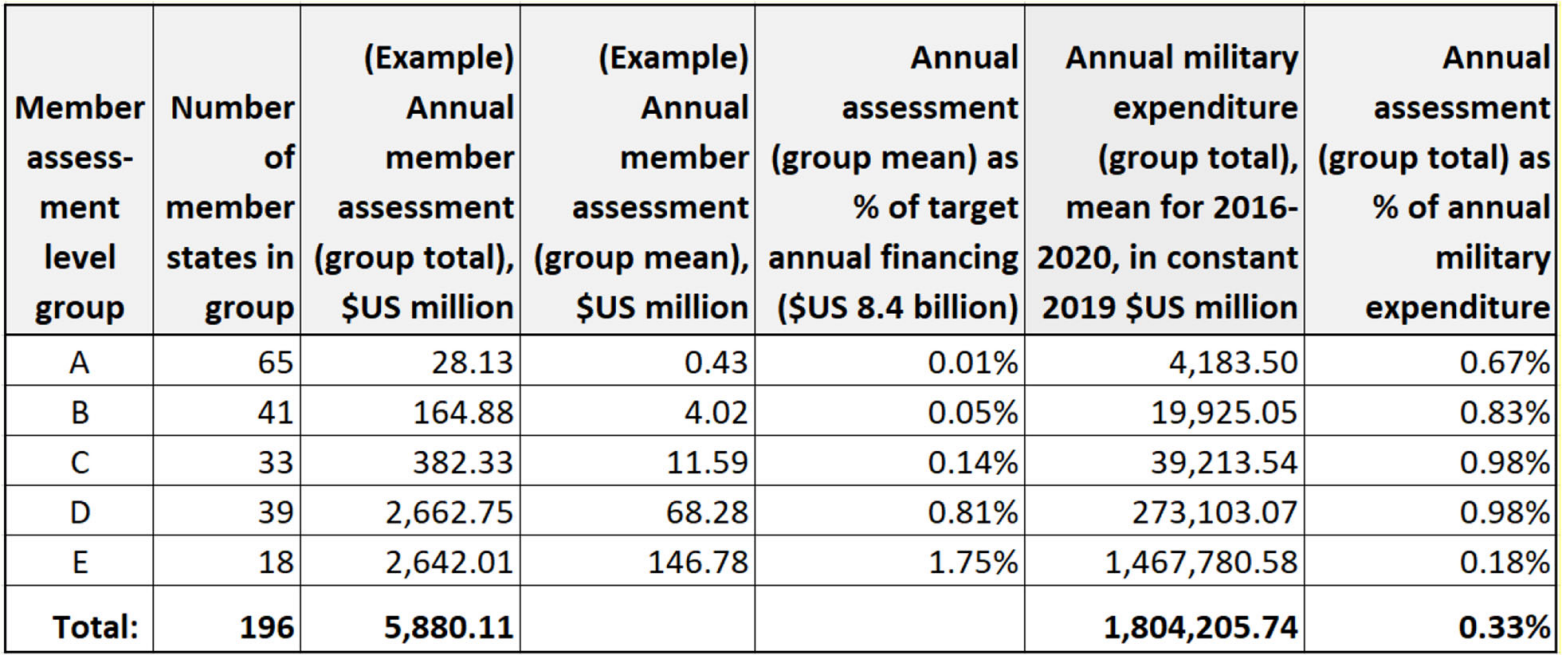
A scenario in which structural integrity of WHO financing is restored, with member assessments greatly increased yet not exceeding 1% of military expenditures *(For illustrative purposes only)*

**Data sources:** See information accompanying Table 1

**Table 2 notes:**

1. Table 2 shows that the proposed increase in assessed contributions, within the recommended limits, can be achieved without burdening members with assessments larger than a small fraction of their annual military expenditure

2. This illustration assumes a WHO financing target of $US 16.8 billion biennially ($US 8.4 billion annually), which is triple the 2018–2019 level. It also assumes that member assessments make up 70% of WHO financing (i.e., $US 5.88 billion annually), and that each member’s assessment is restricted to no more than 2% of total financing (i.e., $US 168 million annually), in order to ensure that financial responsibility is equitably distributed. (Refer to recommendations 1, 2, and 4 for details.) In addition, the illustration adheres to an arbitrary constraint that the mean member assessment in each group (A-E) will be no greater than 1% of the group’s mean military expenditure

3. For optimal clarity, this example does not include voluntary contributions from member states or external partners, which would make up the remaining 30% of total WHO financing in this scenario

We acknowledge that the proposed structural changes in WHO financing would be insufficient to achieve the desired strengthening of the agency if not accompanied by robust procedural changes, appropriate checks and balances within and across the organization’s financial management and administrative functions, and especially, a concerted will to persevere on a path of change. Although the inner workings of the WHA and WHO are beyond the scope of our recommendations, we believe that—if there is political will—careful attention to systemic reforms consistent with the recommendations discussed here will be possible and necessary. Other writers have discussed a number of such opportunities for reform, addressing concerns including transparency and accountability, efficiency, decentralization, and appropriate delineation of core and supportive functions [4, 10].

## Conclusions

The WHO faces enormous challenges, many of which are inherent in the interplay among humans, animals, microbes, and the environment. Over the past seven decades of the WHO’s existence, the agency’s scope of responsibility has steadily increased; yet since the 1980s the level of financial support through assessments to member countries, originally intended to support its core functions, has been frozen. During the same period, voluntary contributions from a handful of members and external donors, including earmarked contributions, have risen dramatically. In our view, this constitutes a structural defect in the financing mechanism. The changes we propose here bring a comprehensive remedy: an increase in total funding; restoration of the primacy of member assessments as the main source of financing; transitioning of most voluntary contributions from the earmarked to the flexible category; and distribution of assessed contributions equitably across all member countries. Importantly, we recommend setting limits on the proportional amount that any individual member country or non-member partner would be permitted to contribute. Taken together, we are confident that these corrections would strengthen the WHO’s finances; ensure sustainability; alleviate concerns about real or perceived influence from powerful members and non-member partners; promote equity among member states; and restore supremacy and autonomy to the WHA. As a contribution to this effort, we offer a simple spreadsheet-based tool that could be used to model the implementation of these changes at a high level and in a stepwise manner, to visualize how the desired outcome might be achieved.

By creating a financial structure in which responsibility was to be equitably distributed among all member states through assessments, the founding WHA was intentional in safeguarding both the agency’s autonomy and member states’ trust. The changes proposed here, we believe, realign the WHO’s financial structure to the founders’ vision. We note also that our recommendations are consistent with the most recent (May 2021) WHA conclusions regarding the urgent need to improve financial sustainability [5]. In contrast to a prevailing assumption that only the wealthiest member states and external partners are in a position to significantly increase their financial contributions, we have shown that most members—regardless of wealth—have experienced two decades of economic growth and that the proposed increase in assessed contributions, within the suggested limits, could be achieved without burdening members with assessments larger than a small fraction of their annual military expenditures. Furthermore, the level of funding we have proposed is miniscule in comparison to the staggering amounts spent by member countries—trillions of dollars so far—to support their citizens and to prevent economic collapse in response to the COVID-19 pandemic. At this time of reflection, many possibilities for strengthening public health infrastructure are undoubtedly being explored. Prioritizing the WHO’s financing and removing the structural defect that limits its leadership role in global health will empower the agency to meet the inevitable challenges of the coming decades.

## Supplementary Information

**Additional file 1.**

**Additional file 2.**

## Data Availability

All data summarized and displayed in Tables 1 and 2 are from publicly available datasets. WHO member assessment and contribution data are from the World Health Organization [7]. Per-capita GDP data for all member countries are from the World Bank Group [16], and military expenditure data for all members are from the Stockholm International Peace Research Institute [17]. Data from these sources were combined into Excel spreadsheets, which are available from the corresponding author on reasonable request. The supplementary material (spreadsheet-based modeling tool) is available from the corresponding author on reasonable request.

## Abbreviations

**COVID-19**: Coronavirus disease 2019
**PHEIC**: Public Health Emergency of International Concern
**SARS**: Severe acute respiratory syndrome
**SARS-CoV-2**: Severe acute respiratory syndrome coronavirus 2
**WHA**: World Health Assembly
**WHO**: World Health Organization
