# Supplementary material for: Strengthening the WHO in the pandemic era by removing a persistent structural defect in financing
Source: Global Health. 2021 Dec 15;17:142. doi: 10.1186/s12992-021-00780-7 (PMC8672333; doi:10.1186/s12992-021-00780-7)
Supplement: Supplementary file 1 — Additional file 1. [file 12992_2021_780_MOESM1_ESM.docx]

**Supplementary material**

***1. Spreadsheet-based modeling tool***

A spreadsheet-based modeling tool (see image below), available on request from the corresponding author, may be used to create a multi-step financing model for gradual adjustment of the financing structure, based on specified aims and contribution limits. This tool works only with high-level categories of financing amounts, not contributions by individual members or donors.


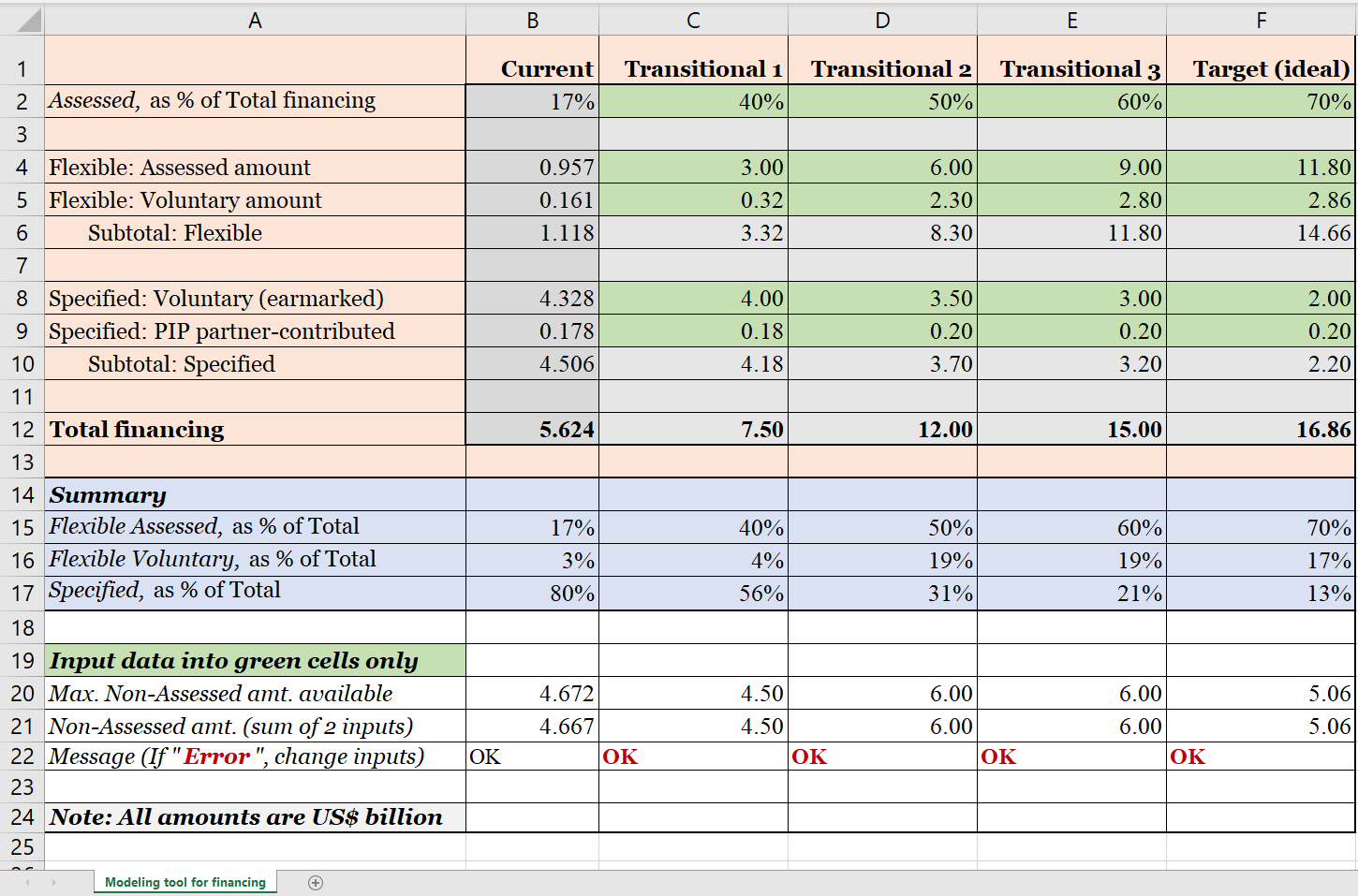


*Instructions*:

Data should be entered into green-colored cells only. The modeler may want to begin by entering values in row 2 for the desired *Assessed* percentage of total funds at each transitional step. As additional entries are made for the 2 *Flexible* and 2 *Specified* amounts, the changing values in the uncolored cells in the area below will assist the modeler to make adjustments until acceptable proportions are attained.

Note: In the example shown above, all amounts are in US$ billion. These results were used to create the graph displayed in Figure 2.
