# Supplementary material for: Strengthening the WHO in the pandemic era by removing a persistent structural defect in financing
Source: Global Health. 2021 Dec 15;17:142. doi: 10.1186/s12992-021-00780-7 (PMC8672333; doi:10.1186/s12992-021-00780-7)
Supplement: Supplementary file 2 — Additional file 2. [file 12992_2021_780_MOESM2_ESM.docx]

**Supplementary material**

***2. WHO members and associate members, grouped by 2022-2023 assessment level***

| **Member Country Group** | **Member assessment level for 2022-2023 biennium (approximately constant for 2 decades)** | **Number of members** |
| --- | --- | --- |
| **A** | Assessment <= $50,000 | 65 |
| **B** | Assessment > $50,000, <= $200,000 | 41 |
| **C** | Assessment > $200,000, <= $1,000,000 | 33 |
| **D** | Assessment > $1,000,000, <= $10,000,000 | 39 |
| **E** | Assessment > $10,000,000 | 18 |
|  | **Total** | **196** |

**Note**: Member assessment level groups (A-E) were created by the authors as a convenient way to summarize data. The data summarized represent 194 WHO members and 2 associate members.

**Group A:**

Andorra, Antigua and Barbuda, Belize, Benin, Bhutan, Burkina Faso, Burundi, Cabo Verde, Central African Republic, Chad, Comoros, Cook Islands, Djibouti, Dominica, Eritrea, Eswatini, Fiji, Gambia, Grenada, Guinea, Guinea-Bissau, Guyana, Haiti, Kiribati, Kyrgyzstan, Lao People’s Democratic Republic, Lesotho, Liberia, Madagascar, Malawi, Maldives, Mali, Marshall Islands, Mauritania, Micronesia, Moldova, Mongolia, Montenegro, Mozambique, Nauru, Nicaragua, Niger, Niue, Palau, Puerto Rico, Rwanda, Saint Kitts and Nevis, Saint Lucia, Saint Vincent and the Grenadines, Samoa, San Marino, Sao Tome and Principe, Seychelles, Sierra Leone, Solomon Islands, Somalia, Suriname, Tajikistan, Timor-Leste, Togo, Tokelau, Tonga, Tuvalu, Vanuatu, Zimbabwe

**Group B:**

Afghanistan, Albania, Angola, Armenia, Bahamas, Bangladesh, Barbados, Bolivia, Bosnia and Herzegovina, Botswana, Cambodia, Cameroon, Congo (Democratic Republic of), Congo (Republic of), Côte d’Ivoire, El Salvador, Equatorial Guinea, Ethiopia, Gabon, Georgia, Ghana, Honduras, Jamaica, Korea (Democratic People’s Republic of), Malta, Mauritius, Monaco, Myanmar, Namibia, Nepal, North Macedonia, Papua New Guinea, Paraguay, Senegal, South Sudan, Sudan, Syrian Arab Republic, Tanzania, Uganda, Yemen, Zambia

*(Continued on next page)*

**Group C:**

Azerbaijan, Bahrain, Belarus, Brunei Darussalam, Bulgaria, Costa Rica, Croatia, Cuba, Cyprus, Dominican Republic, Ecuador, Estonia, Guatemala, Iceland, Jordan, Kenya, Latvia, Lebanon, Libya, Lithuania, Luxembourg, Morocco, Panama, Serbia, Slovenia, Sri Lanka, Trinidad and Tobago, Tunisia, Turkmenistan, Ukraine, Uruguay, Uzbekistan, Vietnam

**Group D:**

Algeria, Argentina, Austria, Belgium, Chile, Colombia, Czechia, Denmark, Egypt, Finland, Greece, Hungary, India, Indonesia, Iran, Iraq, Ireland, Israel, Kazakhstan, Kuwait, Malaysia, New Zealand, Nigeria, Norway, Oman, Pakistan, Peru, Philippines, Poland, Portugal, Qatar, Romania, Singapore, Slovakia, South Africa, Sweden, Thailand, United Arab Emirates, Venezuela

**Group E:**

Australia, Brazil, Canada, China, France, Germany, Italy, Japan, Korea (Republic of), Mexico, Netherlands, Russian Federation, Saudi Arabia, Spain, Switzerland, Turkey, United Kingdom, United States of America
